# Development of a tailored, telehealth intervention to address chronic pain and heavy drinking among people with HIV infection: integrating perspectives of patients in HIV care

**DOI:** 10.1186/s13722-019-0165-1

**Published:** 2019-08-29

**Authors:** Tibor P. Palfai, Jessica L. Taylor, Richard Saitz, Maya P. L. Kratzer, John D. Otis, Judith A. Bernstein

**Affiliations:** 1 Department of Psychological and Brain Sciences, Boston University, 900 Commonwealth Ave., Boston, MA 02215 USA; 2 Clinical Addiction Research and Education (CARE) Unit, Section of General Internal Medicine, Department of Medicine, Boston Medical Center and Boston University School of Medicine, 801 Massachusetts Ave, Boston, MA USA; 3 Department of Community Health Sciences, Boston University School of Public Health, 801 Massachusetts Ave, Boston, MA USA; 4 Grayken Center for Addiction, Boston Medical Center, Boston, MA USA

**Keywords:** HIV, Chronic pain, Alcohol, Heavy drinking, Self-management

## Abstract

**Background:**

Chronic pain and heavy drinking commonly co-occur and can influence the course of HIV. There have been no interventions designed to address both of these conditions among people living with HIV (PLWH), and none that have used telehealth methods. The purpose of this study was to better understand pain symptoms, patterns of alcohol use, treatment experiences, and technology use among PLWH in order to tailor a telehealth intervention that addresses these conditions.

**Subjects:**

Ten participants with moderate or greater chronic pain and heavy drinking were recruited from a cohort of patients engaged in HIV-care (Boston Alcohol Research Collaborative on HIV/AIDS Cohort) and from an integrated HIV/primary care clinic at a large urban hospital.

**Methods:**

One-on-one interviews were conducted with participants to understand experiences and treatment of HIV, chronic pain, and alcohol use. Participants’ perceptions of the influence of alcohol on HIV and chronic pain were explored as was motivation to change drinking. Technology use and treatment preferences were examined in the final section of the interview. Interviews were recorded, transcribed and uploaded into NVivo^®^ v12 software for analysis. A codebook was developed based on interviews followed by thematic analysis in which specific meanings were assigned to codes. Interviews were supplemented with Likert-response items to evaluate components of the proposed intervention.

**Results:**

A number of themes were identified that had implications for intervention tailoring including: resilience in coping with HIV; autonomy in health care decision-making; coping with pain, stress, and emotion; understanding treatment rationale; depression and social withdrawal; motives to drink and refrain from drinking; technology use and capacity; and preference for intervention structure and style. Ratings of intervention components indicated that participants viewed each of the proposed intervention content areas as “helpful” to “very helpful”. Videoconferencing was viewed as an acceptable modality for intervention delivery.

**Conclusions:**

Results helped specify treatment targets and provided information about how to enhance intervention delivery. The interviews supported the view that videoconferencing is an acceptable telehealth method of addressing chronic pain and heavy drinking among PLWH.

## Background

Heavy drinking among people living with HIV/AIDS (PLWH) has direct effects on HIV-related symptoms and indirect, deleterious effects on HIV outcomes through non-adherence to care recommendations [1]. These considerations have led to recent efforts to integrate alcohol interventions into HIV-care. Brief interventions that emphasize motivational interviewing have been examined in HIV-care settings [2–4]. Although these interventions have shown some promise, the beneficial effects on drinking outcomes have been limited and have suggested that a more intensive approach may be required to motivate and maintain change in alcohol use [2], particularly among those with significant comorbid conditions [5]. One of the more important of these comorbid conditions is chronic pain [6, 7]. PLWH report high rates of chronic pain, which exceed 50% in some HIV-clinic cohorts [6, 7]. The associations between chronic pain, heavy alcohol use, and HIV/AIDS have been described as complex and multidirectional [8], with impacts on medication adherence [9, 10], immune system efficiency [11], disease progression [12], depression and anxiety [13], and sensitivity to pain [14]. Rates of chronic pain are higher among those who engage in heavy drinking and chronic pain has a negative impact on alcohol outcomes [15, 16]. Among PLWH, moderate to severe chronic pain has been linked to increased risky drinking over time [8]. Behavioral interventions have been shown to be effective for pain management [17] but there have been few efforts to tailor approaches to the unique needs and characteristics of HIV-populations (see Merlin et al. [18] for an exception). Similarly, despite the development of strategies to address heavy alcohol use among PLWH [2, 3], thus far, no intervention has been developed to address the highly comorbid conditions of chronic pain and heavy drinking together among PLWH.

Even with the development of efficacious behavioral treatments, attendance to clinic visits represents a substantial barrier for PLWH who experience pain and alcohol/substance use. High rates of drop-out and missed sessions, common in community-based pain management in-person interventions, are a considerable concern for PLWH [19] who may experience additional burdens related to HIV symptoms and other co-occurring conditions, financial barriers, and stigma related to alcohol and substance use [20]. Finding alternative modalities to deliver integrated, efficacious behavioral treatments that reduce barriers associated with clinic attendance may improve outcomes.

The purpose of this study was to learn about the associations among HIV/AIDS, pain and heavy drinking among patients in HIV-care in order to tailor a videoconferencing intervention for chronic pain and heavy drinking. We chose videoconferencing as our platform because studies across a number of disorders have shown its advantages for improving adherence particularly among populations that face significant barriers to treatment, such as low-income populations [21–23]. Videoconferencing has a distinct advantage over many other forms of telehealth in that it provides the interventionist with real time information about how patients are able to utilize skills and provides the capacity to provide synchronous training and feedback. It also provides a platform to introduce more extensive technology enhancements (e.g., web-based assessments, experience sampling, video-skills training) for both clinical and research purposes [21, 24]. A number of studies have shown that video telehealth interventions are equivalent to in-person sessions in terms of patient satisfaction with treatment [25, 26]. Based on previous work on cognitive-behavioral treatment for pain [27], self-management approaches for alcohol use [28, 29], pain management [18, 30], and alcohol use [2, 3] an initial integrated intervention addressing the anticipated treatment needs of the study population was designed. The first intervention component was designed to help patients understand the role of various lifestyle factors in the experience of pain and increase readiness-to-change alcohol use through motivational interviewing strategies. This and subsequent modules included discussion of how pain and alcohol use were associated with HIV (e.g., influence of drinking on HIV medication adherence, HIV-related pain, etc.). Subsequent behavioral components relevant to both pain and heavy drinking were addressed including behavioral activation, functional analysis, stress and coping, automatic thinking and cognitive restructuring, and sleep hygiene. Patients also learned strategies for behavioral pacing related to pain and alcohol-related harm reduction strategies.

Individual participant interviews were conducted to: (1) determine the utility and importance of the various content areas of the proposed intervention and the use of the telehealth modality (videoconferencing), (2) understand participant experiences of chronic pain and patterns of alcohol use, (3) gain insight into health care experiences that participants found helpful and not helpful related to HIV, pain and alcohol use, (4) identify potential barriers and facilitators of intervention adherence, (5) clarify the use of various technologies and preferences for intervention modalities, and, most importantly (6) learn about content and process features that may be important to include in the technology-based intervention.

## Methods

### Design

In this study, a semi-structured, in-person interview was administered by a clinical psychologist (White, non-Hispanic, male with 20+ years of clinical experience) to: (1) elicit participant feedback regarding the proposed content and structure of the technology-based intervention, (2) extract themes that could be used to tailor intervention content and structure, and (3) develop a better understanding of smartphone and internet technology use in this population to ascertain the acceptability of videoconferencing as a modality of delivering the technology-based intervention.

### Participants

Participants were eligible if they were 18 years of age or older, fluent in English, had documented HIV infection in the medical record, reported at least 3 months of non-cancer related pain (defined as moderate or greater pain in the past week) and exceeded US recommended limits for risky drinking: at least 1 or more heavy drinking episodes in the past month (≥ 4 standard drinks on one occasion for women and ≥ 5 for men) or exceeded weekly limits (> 7 for women/> 14 for men). Participants currently using pharmacological approaches to manage either pain or alcohol use were permitted if medication doses were stable (i.e. same prescribed dose for at least 2 months). Participants with a history of bipolar disorder, schizophrenia, or complicated alcohol withdrawal (i.e. delirium tremens or withdrawal seizure), those in current psychosocial treatment for pain or alcohol use, and those with an anticipated surgery in the next 6 months were excluded.

### Recruitment

Participants were recruited from the Boston Alcohol Research Collaborative on HIV/AIDS Cohort (Boston ARCH Cohort), a component of the Consortia for HIV/AIDS and Alcohol-Related Research Trials (CHAART) following study visits. Inclusion criteria for the Boston ARCH study were documentation of HIV infection in the medical record, current or past 12-month drug or alcohol dependence (based on DSMIV criteria) and/or ever injection drug use, fluency in English, and age 18 years or older [31]. Participants were also recruited from a hospital-based HIV/primary care clinic at a large, urban academic tertiary setting by clinician referral. Participants were screened for the study in-person or by telephone. Of the Boston ARCH Cohort, 60 participants were approached for screening, 50 participants completed screening, 10 were eligible, and 9 agreed to participate in the interview study. From the clinic sample, one participant was referred, screened, and enrolled in the study. Enrollment of new participants was terminated when interviews reached data saturation (the point at which content was both rich in quality and thick in quantity, and no new information emerged) [32].

### Data collection

A semi-structured interview schedule was developed using a Delphi process and a panel with expertise in areas of interest: infectious disease management, pain management, unhealthy drinking, and techniques for intervention based on cognitive behavioral therapy. A one-on-one, 50-min interview was then conducted by a clinical psychologist to elicit participant experiences of pain, alcohol use, and treatment within the context of HIV (see Table 1 for key interview probes). Participants shared their experiences with HIV and HIV care, followed by a discussion about pain duration, interference, triggers and coping strategies. This included medical and psychological approaches to coping and social supports. A discussion of alcohol use patterns followed, including the contexts in which alcohol was most often used, the role of alcohol in pain management, and effects of alcohol on HIV management or medication and treatment adherence. Perspectives on the need and ability to change alcohol use were also examined during this section.

**Table 1:** Interview guide and key probes

Experiences with HIV and HIV care	
HIV history	To start, would you mind talking with me a little bit about your experience with HIV? When did you receive your diagnosis? (Probe: When were you told by a health care provider that you had HIV?)
HIV current status	What is your understanding of your HIV status currently? (Probe: How well is your HIV being managed medically?) How do you cope with and manage your symptoms?
HIV management for future	When you think about the future, how do you imagine you will be managing your HIV? What do you see as the main challenges for managing your HIV symptoms in the future?
Understanding pain experience and treatment	
Description of pain and interference	Could you tell me a little bit about the sort of chronic pain you have had? What is it like? When does it get better? What makes it worse? How does the pain interfere with your life? How would your life be different if your pain was reduced or better managed?
Pain treatment and coping	What do you do to cope with or manage your pain? (Probe: How do you get through the bad times with your pain?) Are you taking any medications for your pain? Is there anything else that you are currently doing to help deal with your pain? (Probe: How does that work for you?)
Social support	Do other people in your life know about your pain? Have they helped you cope or made it more difficult? (Probe: In what way?)
Alcohol and substance use	
Patterns of use and contexts	I would like to get a sense from you about how alcohol fits in with your life. When do you drink? How many days per week? How much do you typically drink? What are the main reasons why you drink? (Probe: If you think back over the last week, what things happened or what were you feeling just before you picked up a drink?)
Drinking and pain	How, if it all, is your drinking related to your pain? (Probe: Do you use alcohol to help your pain? How so?)
Alcohol and medication	How is drinking related to your use of medications: HIV meds, pain meds, other medications? (Probe: Do you tend to drink with medications? What about forgetting to take them?)
Alcohol-related consequences	Are there any negative effects of drinking for you? [Probe: Any not so good things, like effects on either your health in general or HIV?)
Experiences with treatment	
Treatment for pain	Have you talked to your doctor about your pain? What have they recommended for you? Have you faced any difficulties getting treatment for your pain? (Probe: If so, what have they been?) Have you ever had anyone talk to you about ways to manage your pain? (Probe: If so, can you tell me about it?) If yes: What did you like and what did you dislike about counseling to help you manage pain? If no: What do you think about having someone to talk to you about things you could do in addition to medication to help you or deal with your pain?
Treatment for alcohol use or other substances	Have you ever had treatment/counseling for drinking? (Probe: Can you tell me about it?) What did you like and what did you dislike about this kind of treatment/counseling? (Probe: What did you get out of your treatment/counseling?) Have you ever had treatment or any help for drug use? What about your treatment for alcohol or substances has been most helpful? (Probe: How were you able to use it to improve your health?)
Technology use and access	
Smartphone access	Do you have a smartphone: a phone that has access to the internet? What kind of phone is it? Do you have a data plan that you use? Do you have unlimited data? For what activities do you usually use your phone?
Internet access	Do you have access to the internet? If yes: How often do you use the internet? Can you use it in a private space? (Probe: Can you be alone when you use it?) If no: Where could you get access to the internet if you wanted to? How difficult/easy would it be to access the internet in this way?
Overview of the program and modalities	
Smartphone and videoconferencing	What do you think of using the video/phone approach to deliver an intervention? What do you see as the main advantages and disadvantages of doing an intervention for your pain and alcohol use by phone or video in this way?
Duration	What do you see as a reasonable number of sessions for working with a counselor on ways to better manage your pain and alcohol use? What do you see as a reasonable amount of time for a session with a counselor to talk about your pain and alcohol use?
Ways to improve	Are there any other ideas that you have about how we could make the intervention better: more useful to you, more interesting for you, more likely that you would want to use it? (Probe: This could mean the content, ways of delivering it, other uses of technology [e.g., text messaging, web]) If you were designing a way to help other people living with HIV who had pain and consumed alcohol, what sorts of things would you want to do?

In the next section, participants were asked to discuss their experiences with different types of treatment for pain, alcohol and substance use and HIV. They were prompted to describe barriers to treatment, aspects of treatment that they liked and disliked, and what they perceived to be helpful and not helpful. This segment ended with a brief discussion of what the participant saw as their priorities among health issues and the type of support that would be most helpful to them in addressing these needs.

In the final section, participants had an opportunity to review and comment on proposed intervention modules and rate them in terms of their perceived usefulness/helpfulness (1 “not at all”—5 “very”). Participants were then asked to provide opinions about the structure of the intervention, including the number and duration of sessions and the use of videoconferencing. This section included questions about technology use, including availability of smartphone and data plan, use and frequency of text messaging, and use and frequency of internet browsing. The final section was devoted to eliciting ideas about ways to improve or modify the intervention based on what would be most helpful to them personally.

### Analytic measures

All interviews were recorded, transcribed, and uploaded into NVivo^®^ v12 software for qualitative analysis. Two study investigators (TP, JB) and an external consultant participated in the process, which began with developing first impression codes independently, comparing them, and arriving by consensus at a reconciled codebook. This was followed by thematic analysis of the data in which specific meanings were assigned by the team to code content retrieved from NVivo. Two coders (TP, JB) conducted the final data coding. In the last stage, themes were sorted according to their potential for tailoring specific intervention components and presented results to the entire study team. Descriptive analysis of Likert format questions about preferences for types of content, formalities, and ease of technology use were performed using SPSS v. 24.

## Results

### Sample characteristics

There were 10 interviews conducted. Descriptive statistics are provided in Table 2. The mean age was 53.3 (SD = 8.8). Seven participants identified as male and eight identified race as Black/African American, while two identified as White. Two identified ethnicity as Hispanic. Participants in this sample had been living with HIV for many years (mean number of years since diagnosis = 19.5 (SD = 5.7), range 12.0 to 28.0 years). All participants had a past history of substance use. Participants experienced moderate to severe chronic pain (mean pain severity rating = 7.3 (SD = 2.1), range of 4.0 to 10.0). All participants were eligible based on heavy episodic drinking with a mean of 8.0 (SD = 7.2) heavy drinking episodes in the past month.

**Table 2:** Sample characteristics

Characteristics	n (%)
Age (year), mean (SD)	53.3 (8.83)
Gender	
Male	7 (70)
Female	3 (30)
Race	
Black or African American	8 (80)
White	2 (20)
Ethnicity	
Non-Hispanic or Latino	8 (80)
Hispanic or Latino	2 (20)
Avg. weekly pain (0–10), mean (SD)	7.33 (2.06)
Drinks per week	19.4 (12.6)
Heavy drinking episodes past month, mean (SD)	8.00 (7.17)

### Intervention themes from qualitative analysis

Nine categories of themes emerged that helped reinforce the value of specific intervention components, provided insight into areas that were of particular importance to address, or pointed to adaptations that would increase acceptability of the intervention or enhance its effectiveness. Themes with direct implications for intervention included: (1) the challenges faced by and the resilience of participants coping with HIV, (2) a strong need for autonomy, (3) the importance of providing a clear rationale for treatment approach and components, (4) the prevalence of depressed affect and behavioral disengagement, (5) the central role of stress and emotional triggers for pain and drinking, (6) factors related to motives to drink and refrain from drinking, (7) technology use, (8) interest in intervention components, and (9) preferences for intervention structure and style. Illustrative statements for each theme are provided below; the set of participant statements corresponding to themes are presented in Table 3.

**Table 3:** Intervention themes and quotes from qualitative analyses

Intervention themes	Quote
1. Resilience in coping with HIV may serve as a basis for self-efficacy for new behavioral change	*“Five or six of those [post*-*diagnosis] years was a struggle with doing things I wouldn’t normally do like drinking, smoking and carrying on… as if nobody gonna treat me the same anymore.” [Participant 28, Black male in his 40’s]* *“So I didn’t let it define me. It was a moment that I needed to make…do you want to get better or do you just want to call it quits?” [Participant 11, Black Hispanic male in his 40’s]* *“I mean to me like, my experience was really heavy, heavy stuff. I don’t know how I was able to get through it, you know? I mean, I guess my faith in God and prayers from my mom and my family.” [Participant 51, White Hispanic male in his 60’s]* *“…So I have to deal with it the best that I can…I don’t let it stop me from doing what I need to do….Just ride the bus until you can’t anymore and they can’t do anything for you.” [Participant 5, Black female in her 60’s]*
2. Importance of autonomy in health care decision-making	*“She asked me do you want to go to AA classes or whatever, or be checked in somewhere and I told her no, I can handle it…I’m trying to get to the point where I could just stop, really. But I want to do it on my own.” [Participant 46, Black female in her 60’s]* *“Bless her heart she tries it because she’s my doctor….What she doesn’t get is these are things that I want to do.” [Participant 32, Black male in his 40’s]* *“Everything has to be after 3* *pm because I work. And I can’t like take a day off here.” [Participant 44, Black male in his 60’s]* *“I’d try it. Like everything, I try. I couldn’t say if I would keep going or not keep going.” [Participant 28, Black male in his 40’s]* *“Sometimes people don’t want to leave the house or come for help.” [Participant 11, Black Hispanic male in his 40’s]*
3. Importance of clarifying the rationale for the intervention approach	*“And they started asking about my father. And I got to told them I’m not here for my father, I’m here for myself.” [Participant 5, Black female in her 60’s]* *“I was going and I just stopped. My doctor always say, “We’re going to set you up with physical therapy.” And I go “okay.” I go for a couple of times and… then that’s it.” [Participant 28, Black male in his 40’s]* *“I tried once but I think I didn’t have, maybe it’s the person. I didn’t have a positive*- *I didn’t receive it positively.” [Participant 42, Black female in her 40’s]* *“I just didn’t get it.”; “They have ideas to remove (pain), but it’s not working. Like example. She asking me to close my eyes, and … put all bad memories inside the jar and close it. Go to ocean and throw it. And open your eyes. I opened my eyes. She said, “How do you feel?” and I told her “terrible.” [Participant 21, White male in his 50’s]* *“I’ve never heard of a lot of pain management. I’ve always thought, “What are they talking about?” No idea what they mean by pain management.” [Participant 13, Black male in his 40’s]*
4. Depression and behavioral withdrawal	*“It’s okay. You know I’m not doing nothing really. It’s just appointments and I don’t work nowhere. I’m disabled.” [Participant 46, Black female in her 60’s]* *“And you just want to crawl in a dark place and no, you don’t want to be bothered.” Participant 11, Black Hispanic male in his 40’s]* *“I’m not from here so I don’t know a lot of people here. And all the people here that I did know, either have died or have moved out of town. So I’m pretty basically in the house.” [Participant 32, Black male in his 40’s]* *HIV prevents me from being around certain people or crowds. Because I know what their minds thinking, you know what I mean….And I wanted to see people treat me the same when after I say that [that I am HIV positive].” [Participant 28, Black male in his 40’s]* *“There are times when I just don’t have the energy to do things…I just don’t want to be a part of society.” [Participant 32, Black male in his 40’s]* *“I don’t have friends. I have acquaintances, I have associates, I don’t have friends.” [Participant 32, Black male in his 40’s]*
5. Pain, stress and emotion coping	*“So I’m tired about the situation. If I will call my doctor, she will tell me this is age or take ibuprofen or Tylenol. Or do exercise… When I get more depressed I have more pain.” [Participant 21, White male in his 50’s]* *“Sometimes I feel I am responsible about this [family health problems]. …This is why [I have] this pain, pain from deep inside and pain in my shoulder and in my head… And so sometimes I drink to forget.” [Participant 21, White male in his 50’s]* *“If I don’t, sometimes the [pain] will trigger and aggravate me. Then I think about the HIV.” [Participant 46, Black female in her 60’s]* *“Because I couldn’t take, I couldn’t face the reality of anything. I was in pain emotionally. I was physically in pain.” [Participant 44, Black male in his 60’s]* *“But you know I got off that [Percocets]. But I still didn’t let my drinking go….Well, maybe I do [drink to cope with pain]. I’m not realizing it.” [Participant 5, Black female in her 60’s]* *“With feelings, my anxiety, my depression, if I feel I’m getting very depressed I drink more. And it helps me, a lot if I think about the HIV, which I try not to think about it.” [Participant 46, Black female in her 60’s]* *“Because of pain I just want to be numb. Sometimes I’m so defeated. A whiskey drink can take the pain away…I just drink not to think.” [Participant 42, Black female in her 40’s]* *“Marijuana helps me manage my depression from the pain.” [Participant 28, Black male in his 40’s]* *“I smoke marijuana. And it subsides everything. It calms the leg and everything. You know I’m just peachy. I just lay down and I’m ok.” [Participant 44, Black male in his 60’s]*
6. Motives to drink and motives to refrain from drinking	*“I don’t want to drink during the week. I don’t want to be an alcoholic and stuff like that….so that’s why…only Thursday Friday Saturday.” [Participant 13, Black male in his 40’s]* *“But I take pain meds for it too. But I take them as needed, I try to take them as less as possible.” [Participant 5, Black female in her 60’s]* *“Sometimes the alcohol you use is just to ease your mind…or you get upset and might get frustrated and you take that cold beer and then you don’t want to hear nothing now.” [Participant 5, Black female in her 60’s]* *“I think people are just coping, trying to shut up that inner voice that’s crying for help and they drown themselves…Sometimes I get too bored, nothing to do.” [Participant 11, Black Hispanic male in his 40’s]* *“You know I can change the beer drinking but at this point, I’m going to be honest, I don’t want to.” [Participant 5, Black female in her 60’s]* *“Timewise, forgetful you know. Because I’m drinking beer and then I may fall out and I’m like, ‘Oh shoot! The medication.’ So yeah, it [drinking] has sort of affected it.” [Participant 44, Black male in his 60’s]* *“I have my meds on top of the table and view in plain sight. So I’m like, ‘Oh yeah, I need to take my meds.’ So I take them right away.” [Participant 11, Black Hispanic male in his 40’s]* *“Buying alcohol for $20, that’s a lot of money.” [Participant 42, Black female in her 40’s]* *“I might drink too much. Too much beer and I can feel like I’m getting lightheaded. Okay, Or I can*—*it feels funny and I said, ‘my blood pressure has to be up.’” [Participant 5, Black female in her 60’s]* *“I’d rather be in my house where I know I can control the things that I do.” [Participant 32, Black male in his 40’s]* *“Oh no, I’m drinking the right amount that a person should drink.” [Participant 11, Black Hispanic male in his 40’s]* *“I’ve been on top of that from day one. They had the cap box.” [Participant 28, Black male in his 40’s]* *“No matter where I am, no matter what I do, I will wake up out of sleep to take my medication.” [Participant 32, Black male in his 40’s]* *“I have two or three friends who come to drink in my apartment. Mostly because I don’t get in trouble with anything after drinking.” [Participant 13, Black male in his 40’s]*
7. Technology use and capacity	*“I feel like that would be something to reach a lot of other people today. I think a lot of people would be willing to do something like this. Instead of coming to the office to meet with somebody like ‘can you just FaceTime me?’” [Participant 28, Black male in his 40’s]* *“Sometimes people don’t want to leave the house or come for help. Why not have an app that the app can actually help you connect with that person. I like that.” [Participant 11, Black Hispanic male in his 40’s]* *“I would love that… as long as they give me time to do it so I can be like at home, and to not do it in the streets… I’d have no problem with that… Schedule it.” [Participant 13, Black male in his 40’s]* *“I always use YouTube when I am at home. I watch videos every day.” [Participant 21, White male in his 50’s]* *“I like it in a video, yeah, something that you could sort of go back to.” [Participant 13, Black male in his 40’s]*
8. Interest and experience with intervention components	*“Sayin’ that it’s connected in some kind of way but I’m not looking at it connected in it that kind of way, maybe I can learn, well maybe it is connected and I never knew this. That would be very helpful to learn.” [Participant 5, Black female in her 60’s]* *“I think if I would’ve known, like if there was stuff like that, more particularly like that, I would’ve been able to cope with a lot of things sooner than later.” [Participant 11, Black Hispanic male in his 40’s]* *“Depression could be a problem. Depression is a big; it needs to be kept in tab, you know?” [Participant 13, Black male in his 40’s]* *“You know. I*- *even though I’m dealing with my HIV problem or whatever but I never figured that it would be linked to something like that, you know. Not my HIV but my pain or whatever, I don’t know. You know it would be interesting to see.” [Participant 5, Black female in her 60’s]*
9. Preference for intervention structure and style	*“They (the caseworker, medical team and the interventionist) should be in close communication, not divulging everything, everything is confidential but keeping an eye on it.” [Participant 11, Black Hispanic male in his 40’s]* *“I need motivation. I need someone to either remind me or call me or push me.” [Participant 44, Black male in his 60’s]* *“We talk on the level where she understands my every part of the need…because she gives me all the right answers I want to hear.” [Participant 46, Black female in her 60’s]* “*I know that it’s our responsibility, like personal responsibility to get the help that we need but sometimes we need that extra help.” [Participant 11, Black Hispanic male in his 40’s]* *“I’ll say something to the doctor and like I will leave the office and I completely forgot. I would get a phone call from the caseworkers saying…I have the paperwork you needed…..to help is to really make sure that everybody in the person’s team, the healthcare team, is informed about all of these things.” [Participant 11, Black Hispanic male in his 40’s]* *“Well, I mean, having someone to… that you can really feel comfortable with them. Again and I, because I said this earlier, that I can talk to about any and everything of my personal well*-*being.” [Participant 44, Black male in his 60’s]* *“They make me feel like they know me.” [Participant 28, Black male in his 40’s]* *“And we were like family, It was so many years together.” [Participant 51, White Hispanic male in his 60’s]*

#### Resilience in coping with HIV may serve as a foundation for self-efficacy for new behavioral change

Participants described considerable challenges with discrimination, stigma, and shame related to the diagnosis and subsequent management of HIV.

*“Five or six of those [post*-*diagnosis] years was a struggle with doing things I wouldn’t normally do like drinking, smoking and carrying on…as if nobody gonna treat me the same anymore.” [Participant 28, Black male in his 40’s]*

Participants expressed pride in their strength and adaptability. Efforts to manage their lives with HIV was a marker of resilience and strength and they continued to face multiple challenges even in the modern era of HIV treatment.

*“So I didn’t let it define me. It was a moment that I needed to make…do you want to get better or do you just want to call it quits?” [Participant 11, Black Hispanic male in his 40’s]*

*“I mean to me like, my experience was really heavy, heavy stuff. I don’t know how I was able to get through it, you know? I mean, I guess my faith in God and prayers from my mom and my family.” [Participant 51, White Hispanic male in his 60’s]*

*Summary* These comments highlight how HIV has challenged participants’ identities and their resources.

#### Importance of autonomy in health care decision-making

Participants wanted to be afforded control over decisions about health behavior change and wanted their views about strategies to be valued.

*“She asked me do you want to go to AA classes or whatever, or be checked in somewhere and I told her no, I can handle it…I’m trying to get to the point where I could just stop, really. But I want to do it on my own.” [Participant 46, Black female in her 60’s]*

*“Bless her heart she tries it because she’s my doctor… What she doesn’t get is these are things that I want to do.” [Participant 32, Black male in his 40’s]*

They wanted control over when and how they engaged with resources.

*“Everything has to be after 3* *pm because I work. And I can’t like take a day off here.” [Participant 44, Black male in his 60’s]*

*Summary* Comments highlighted the importance of flexibility, and participant participation about decisions regarding treatment.

#### Importance of clarifying the rationale for the intervention approach

Comments regarding previous experiences with behavioral and medical treatments suggested that participants often did not perceive the value or rationale for the treatments provided, and thus they were not interested in adopting or maintaining them.

*“I’ve never heard of a lot of pain management. I’ve always thought, “What are they talking about?” No idea what they mean by pain management.” [Participant 13, Black male in his 40’s]*

*“I just didn’t get it […] they have ideas to remove [pain], but it’s not working. Like example. She asking me to close my eyes, and … put all bad memories inside the jar and close it. Go to ocean and throw it. Open your eyes. I opened my eyes. She said, ‘how do you feel?’ and I told her, ‘terrible.’” [Participant 21, White male in his 50’s]*

#### Depression and behavioral withdrawal

Although it was expected that participants would show elevated levels of depressed affect, the interviews helped emphasize the salience of low positive affect and behavioral disengagement in participant’s lives. Participants remarked on the constriction of activities and social contacts that contribute to their sense of isolation.

*“It’s okay. You know I’m not doing nothing really. It’s just appointments and I don’t work nowhere. I’m disabled.” [Participant 46, Black female in her 60’s]*

*“And you just want to crawl in a dark place and no, you don’t want to be bothered.” Participant 11, Black Hispanic male in his 40’s]*

#### Coping with pain, stress and emotion

Participants recognized a variety of specific triggers for pain, mentioning rain or cold weather [Participants 5, 28], sitting for a long time [Participant 11], staying home [Participant 28], and standing all day [Participant 44]. Central among these were stress and emotional triggers [Participant 44].

“*So I’m tired about the situation. If I will call my doctor, she will tell me this is age or take ibuprofen or Tylenol. Or do exercise… When I get more depressed I have more pain.” [Participant 21, White male in his 50’s]*

Some mentioned that they had learned to deal with it, while others noted that they use alcohol and other substances for relief from stress, pain and negative emotion.

*“Marijuana helps me manage my depression from the pain.” [Participant 28, Black male in his 40’s]*

*“Because of pain I just want to be numb. Sometimes I’m so defeated. A whiskey drink can take the pain away […] I just drink not to think.” [Participant 42, Black female in her 40’s]*

#### Motives to drink and motives to refrain from drinking

Beer was thought to be a harmless alternative to use of opioids for pain management and an acceptable way to relieve stress, and it was considered safe to drink heavily as long as it was only a few days per week.

*“I don’t want to drink during the week. I don’t want to be an alcoholic and stuff like that….so that’s why…only Thursday Friday Saturday.” [Participant 13, Black male in his 40’s]*

*“But I take pain meds for it too. But I take them as needed, I try to take them as less as possible.” [Participant 5, Black female in her 60’s]*

*“So I had to dumb it down a little bit and go to my beers and leave the hard alcohol alone […] I don’t think my body could take that anymore.” [Participant 28, Black male in his 40’s]*

Consistent with the above, participants reported a number of emotion triggers for drinking such as depression and boredom.

*“Sometimes the alcohol you use is just to ease your mind…or you get upset and might get frustrated and you take that cold beer and then you don’t want to hear nothing now.” [Participant 5, Black female in her 60’s]*

*“I think people are just coping, trying to shut up that inner voice that’s crying for help and they drown themselves… Sometimes I get too bored, nothing to do.” [Participant 11, Black Hispanic male in his 40’s]*

Generally speaking, participants described low motivation to change their alcohol use patterns.

*“You know I can change the beer drinking but at this point, I’m going to be honest, I don’t want to.” [Participant 5, Black female in her 60’s]*

There was a general lack of knowledge or concern about the effect of heavy alcohol use on HIV progression but some did recognize its potential effect on medication adherence.

*“Timewise, forgetful you know. Because I’m drinking beer and then I may fall out and I’m like, ‘Oh shoot! The medication.’ So yeah, it [drinking] has sort of affected it.” [Participant 44, Black male in his 60’s]*

However, participants placed high priority on getting and staying healthy and taking medications.

*“I have my meds on top of the table and view in plain sight. So I’m like, ‘Oh yeah, I need to take my meds.’ So I take them right away.” [Participant 11, Black Hispanic male in his 40’s]*

Participants identified specific negative consequences of drinking that might be considered in the context of goals and values that are incongruent with heavy drinking. These included themes related to work, money, family and health (including HIV).

*“Buying alcohol for $20, that’s a lot of money.” [Participant 42, Black female in her 40’s]*

*“I might drink too much. Too much beer and I can feel like I’m getting lightheaded. Okay, Or I can*—*it feels funny and I said, ‘my blood pressure has to be up.’” [Participant 5, Black female in her 60’s]*

Participants identified strategies that they were currently using to prevent alcohol-related harm, primarily through reducing exposure to risk environments.

*“I’d rather be in my house where I know I can control the things that I do.” [Participant 32, Black male in his 40’s]*

*Summary* There were a number of comments in the interviews that provided insight into the goals and values that participants identified as incongruent with alcohol use. Participants identified medication adherence, HIV progression, and health more generally as factors that may contribute to readiness to change drinking.

#### Technology use and capacity

The interviewer asked participants about how they used smartphones and computers and elicited their opinions about the perceived value of videoconferencing as a way of interacting with a provider. For many participants, the use of videoconferencing was familiar as they used phone video capacities (e.g., FaceTime) to connect to friends and relatives. Unlimited data plans were common, and there was consistent use of texting but less web or computer use. Participants were enthusiastic about the potential to have sessions through videoconferencing. The notion of using video segments to supplement sessions was also well received as participants reported frequent use of the smartphone to watch videos.

*“I feel like that would be something to reach a lot of other people today. I think a lot of people would be willing to do something like this. Instead of coming to the office to meet with somebody like ‘can you just FaceTime me?’” [Participant 28, Black male in his 40’s]*

*“Sometimes people don’t want to leave the house or come for help… Why not have an app that the app can actually help you connect with that person.” [Participant 11, Black Hispanic male in his 40’s]*

*“I like it in a video, yeah, Something that you could sort of go back to.” [Participant 13, Black male in his 40’s]*

*Summary* Videoconferencing appears to be a feasible and well-received modality that could be supplemented with additional media such as video segments to reinforce learning. This is a modality that is familiar to participants and readily accessible.

#### Interest and experience with intervention components

In the structured component of the interview, participants provided feedback about experiences and interest in different aspects of the intervention including insight into what information might be most helpful. Participants were asked to rate the usefulness of the various content modules (e.g., behavioral activation, activity pacing) that were proposed for the intervention.

Comments ranged from:* “It’s a good idea”; “I would be interested because I need to find ways to improve myself”; “I think it would be awesome”; I would give it a try, why not”;* “*Nice to open your mind to other things”;* to “*Depends on how useful it is.*”

Regarding psychoeducation about pain, alcohol, HIV associations: *“Sayin’ that it’s connected in some kind of way but I’m not looking at it connected in it that kind of way, maybe I can learn, well maybe it is connected and I never knew this. That would be very helpful to learn.” [Participant 5, Black female in her 60’s]*

Regarding the use of adjunct video materials: *“I think if I would’ve known, like if there was stuff like that, more particularly like that, I would’ve been able to cope with a lot of things sooner than later.” [Participant 11, Black Hispanic male in his 40’s]*

Regarding intervention features to keep in mind: *“Depression could be a problem. Depression is a big; it needs to be kept in tab, you know?” [Participant 13, Black male in his 40’s]*

*Summary* The comments suggested that the participants were receptive to the content and modalities proposed including adjuncts to the intervention such as video clips to help them learn skills. Comments suggested particular interest in learning more about the association between alcohol, pain, and HIV and a recognition of the importance of addressing depressed mood as part of the intervention.

#### Preference for intervention structure and style

Participants made a number of comments about the characteristics of interventions that have been helpful for them in the past, including the importance of trust, empathy and efforts by the care team to seek out the participant to maintain engagement. Participants supported the idea of communication between the interventionist and the health care team, as long as they could be sure of protection of confidentiality within the health care team.

*“They (the caseworker, medical team and the interventionist) should be in close communication, not divulging everything, everything is confidential but keeping an eye on it.” [Participant 11, Black Hispanic male in his 40’s]*

*“I need motivation. I need someone to either remind me or call me or push me.” [Participant 44, Black male in his 60’s]*

Participants valued respect and empathy from their support team above concerns about structure and style, but mentioned a preference for sessions once a week, less than an hour, and scheduled around other obligations such as work.

*“We talk on the level where she understands my every part of the need…because she gives me all the right answers I want to hear.” [Participant 46, Black female in her 60’s]*

*Summary* Participants were very responsive to health care workers who exhibited high empathy and concern, including following up on missed appointments.

### Intervention component ratings

At the end of the interview, participants were asked to rate the perceived usefulness of the various content areas proposed for the intervention based on a brief description of each. Each content area was rated from 1 (“not at all” useful/helpful) to 5 (“very” useful/helpful) using Likert-type items. These Likert-response ratings of the content areas showed a generally positive response to intervention components. Mean ratings for the different content modules were as follows: learning about personal triggers and breathing exercises 4.22 (SD = 0.83); learning ways to manage negative thoughts 4.29 (SD = 1.25); psychoeducation about pain, alcohol and HIV 4.63 (SD = 0.74); behavioral activation [pleasant activities] 4.75 (SD = 0.46); addressing sleep and becoming more active 4.86 (SD = 0.38); managing stress, anxiety and other pain triggers 4.86 (SD = 0.38); learning pacing and alcohol-related harm reduction strategies 4.89 (SD = 0.33); learning ways to continue self-management after treatment was completed 5.0 (SD = 0.0). Participants were also asked whether they would like an adjunct to treatment in the form of a website with information and tips about alcohol and pain management and rated this as 4.29 (SD = 0.76) on a scale from 1 “(dislike very much”) to 5 (“like very much). Thus, ratings of each of the intervention sessions suggested that participants viewed the content developed for the intervention as useful or helpful to them. These ratings were consistent with participant comments in the interviews regarding the importance of addressing domains such as stress, reduced activity, and behavioral withdrawal.

## Discussion

Although chronic pain is common among PLWH, there are few behavioral intervention approaches designed for this population [30, 33] and no intervention, to our knowledge, has been developed to address both chronic pain and heavy drinking for PLWH. This study sought to gain insight from semi-structured interviews with patients in HIV care about how best to develop and deliver an intervention to improve chronic pain management and reduce heavy drinking. Moreover, the goal of these interviews was to improve understanding of how patients used and experienced technologies to inform the delivery of interventions that reduce barriers to care. Through qualitative analyses of these interviews, we were able to develop a patient-informed perspective on how to modify, integrate, and deliver an intervention to reduce heavy drinking and help patients better manage chronic pain. Results provided insight into the importance of different content areas, the potential value of intervention strategies, and the type of therapeutic climate that would maximize patient engagement and behavior change.

We initially constructed a working draft of an intervention tailored to patients in HIV-care based on evidence indicating high rates of functional impairment from pain, high rates of depressive symptoms, stigma and discrimination associated with HIV status [30], and elevated rates of current and prior heavy drinking and substance use [4, 5]. In addition, as these participants were not specifically seeking specialty treatment for alcohol use, we anticipated that enhancing motivation to change alcohol use would be an important intervention target consistent with populations who undergo screening and brief intervention in outpatient medical settings [2–4].

The depressive symptoms and social withdrawal observed in this study was consistent with previous qualitative research on PLWH with chronic pain [18]. Many participants experienced significant social isolation, engaged in few activities, and spent little time outside of the home. The factors that contribute to depressed affect are manifold but clearly the fear of rejection and beliefs about others’ reactions to their HIV status contributed to concerns. Stigma was highly salient to participants in the interviews and exacerbated by other stigmatizing conditions such as chronic pain and alcohol or substance use [18]. Comments from the interviews clarified the importance of directly addressing depressive symptoms such as self-blame, low self-efficacy, withdrawal, and low positive affect in the initial phases of the intervention. Results from the interviews also highlighted the need to find ways to increase pleasant activities among patients, particularly strategies to help patients establish and re-engage with social networks. Behavioral activation is a particularly valuable strategy for addressing depressive symptoms among those experiencing chronic pain [27] and has been utilized with PLWH specifically [30]. This treatment component also provides a way of developing non-alcohol/substance related alternatives for promoting positive affect. A number of studies have shown the value of developing non-substance related alternative reinforcers in patients’ lives for reducing problem drinking [34–36]. As such, the behavioral activation module serves an important dual purpose for addressing negative emotional components of pain and providing alternative sources of positive reinforcement to alcohol use.

The role of emotional stress triggers on chronic pain was also readily identified among participants in this sample consistent with previous work [37]. Participants often identified negative emotions and stress as triggers for chronic pain. Alcohol also served as a way of managing negative emotions for some [6], helping highlight the potential value of a treatment component to help patients address both heavy drinking and chronic pain with more effective affect management strategies. By providing the patient with more effective ways to cope with stress and emotion triggers, one may decrease drinking even among those who do not have an explicit goal of reducing their alcohol use.

Participant comments related to alcohol use provided insight into potential ways to address heavy drinking in this population. Generally, participants did not identify their alcohol use as a point of concern and some stated explicitly that they intended to maintain current use patterns. However, participants identified a number of important goals, values, and concerns (e.g., health, family) that were viewed as inconsistent with heavy drinking. These, particularly health concerns, might be useful to highlight and discuss in efforts to enhance motivation to change [35, 38]. Information about the impact of alcohol on medication adherence and HIV progression for instance, aligns with the priority that participants placed on HIV management and may thus contribute to increased readiness to change.

There were additional comments in the interviews that may be used to inform the alcohol specific component of the intervention. Participants were generally unaware how heavy drinking, chronic pain and HIV symptoms may be related and did not know what levels of alcohol use constitute risk. Psychoeducation to correct misconceptions about standard drinks and provide information about potential risks of drinking to health outcomes may encourage patients to think more about their current alcohol use given the expressed interest in health. Many participants did report the use of strategies to minimize alcohol-related harm even if they did not identify them as such. To maximize engagement and utilization, it may be useful to anchor the discussion of alcohol harm reduction strategies in the context of current strategies that patients use to help keep themselves safe.

By beginning our interview with a discussion of HIV experiences, we were able to appreciate the broader impact of living with HIV on current stressors and coping strategies, motives to use and limit alcohol use, and resilience. It was important to understand participant perspectives on the points of intersection between HIV, alcohol use, and pain and how these conditions had shaped their identities and behavioral choices over time. Participant remarks suggest that the interventionist may foster collaboration by appreciating the ways that HIV has challenged patients’ identity and resources and affirming patients’ resilience where possible. The interventionist may enhance patient engagement and self-efficacy for current intervention objectives by recognizing and drawing upon the patients’ capacity for HIV-related coping and behavior change. It should be noted that participants in this sample were older and had been in stable HIV care for a long time. Specific symptoms of pain, drinking patterns, and concerns about HIV were likely different than patients with new diagnoses of HIV.

The results of the interviews also provided valuable insights into how to structure and modify the proposed modules of the intervention and address key intervention objectives. First, it will be critical to provide clear and explicit rationales for the approach and content areas that comprise this treatment. Participants had varied and unsuccessful experiences with general psychotherapy approaches to pain which led to some skepticism about the value of treatment. Similarly, descriptions of past unsuccessful and brief experiences with physical therapy from participants highlighted the importance of setting realistic expectations about how this approach to pain management might be helpful. It is critical to set expectations for the course of treatment, the expected roles of patient and interventionist, and what outcomes might be expected over what timeframe. As patients may not have experience with behavioral approaches to change, it will be important to explain that the intervention requires practice and sustained involvement to gradually reduce pain interference and control of pain intensity rather than an immediate and significant impact on pain severity. This emphasis on rationale and expectations should be considered throughout the duration of the intervention and provided for each of the content domains with reminders and encouragement.

Second, the importance of patient autonomy and flexibility in the treatment process were clear from participant comments. Participants expressed a strong desire to have choice in the intervention process, including selection of times and circumstances in which they receive information. There may be benefit to offering patients a menu of resource options to permit them some flexibility to modify the intervention approach to fit their lives and current concerns. A related consideration is the critical role of empathy, acceptance, and intervention efforts that demonstrate care and concern. Although the provider-patient relationship is critical to any intervention, they may be particularly important to patients who have faced many years of discrimination and stigma related to HIV. Participants were very responsive to health care workers who exhibited high empathy and concern. This included expressed statements of caring as well as efforts to ensure that the participant stayed engaged in treatment including following up on missed appointments. This intervention must involve particular attention to establishing a collaborative relationship and would likely benefit from a more active strategy to address missed appointments (e.g., more frequent reminders, reaching out to patient to reschedule) than is typical in behavioral practice.

Finally, the interviews provided significant information about technology use and interest in this cohort. Unexpectedly, all participants had their own smartphones, many with unlimited data plans. In contrast, few had ready, private access to a computer. This made it clear that any videoconferencing approach would need to take place over personal smartphones. The videoconferencing approach was very well received by participants who liked the convenience and flexibility of this approach. Participants had sufficient experience through the regular use of video technology to be able to understand and feel confident about using the videoconferencing procedures described.

The interview supported the acceptability of using videoconferencing to reach patients and suggested the possibility of other technologies that might be considered to support the intervention and intervention delivery. The use of videos and other learning materials (e.g., example of homework exercises) may supplement video-conferencing intervention content and be delivered through smartphones. A variety of administrative supports could also be provided through the use of smartphone technology including reminders for appointments and homework scheduling. The ready use of text messaging and use of apps makes it feasible to incorporate these elements to foster engagement. The technology components proposed in the current study provides the potential to automate a number of features of the intervention to ensure that provider time can be used to maximal benefit (e.g., as opposed to reminding patients to do specific tasks), avoid redundancy in service delivery, and promote accessibility and optimal flexibility for patients who wish to use the intervention. The next step will be to pilot test these intervention components among patients recruited from HIV-care to discern acceptability, preferences for technology features, and feasibility prior to an efficacy trial.

Although not part of the current intervention, delivery of the intervention through a health technology platform opens the possibility for a range of additional adjunct components delivered through social media. Given the frequent experience with social withdrawal among HIV patients and stated preferences for group interactions identified in previous work [18], investigators may consider the use of social media and online platforms as additional ways to extend the impact of the intervention by incorporating peer support [39] and other peer-led components [40]. Development of these and other technology-based components will require that investigators are mindful of digital literacy and utilization of specific HIV-populations as they seek to craft mobile health solutions for chronic health conditions [41–43].

## Conclusion

In sum, this study shows how formative qualitative research that identifies themes specific to our target population can have implications for tailoring a novel mobile health intervention to address the confluence of HIV/AIDS, chronic pain and unhealthy alcohol use and testing its efficacy in a randomized controlled trial. These points might have been missed without the inclusion of interviews with patients as the first step in this research agenda. Attention to the identified themes has the potential to increase both patient engagement and motivation to change by addressing specific priorities, matching intervention modalities with patient preferences, and building on lessons from participants’ past experiences with illness and health care delivery.

## Data Availability

Data supporting the analyses are provided in the table in transcribed form.

## Abbreviations

PLWH: people living with HIV/AIDS
Boston ARCH Cohort: Boston Alcohol Research Collaborative on HIV/AIDS Cohort
CHAART: Consortia for HIV/AIDS and Alcohol-Related Research Trials
